# Estimating the Risk for Secondary Cancer After Targeted α-Therapy with ^211^At Intraperitoneal Radioimmunotherapy

**DOI:** 10.2967/jnumed.121.263349

**Published:** 2023-01

**Authors:** Erik Leidermark, Andreas Hallqvist, Lars Jacobsson, Per Karlsson, Erik Holmberg, Tom Bäck, Mia Johansson, Sture Lindegren, Stig Palm, Per Albertsson

**Affiliations:** 1 Region Västra Götaland, Sahlgrenska University Hospital, Gothenburg, Sweden;; 2 Department of Radiation Physics, Institute of Clinical Sciences, Sahlgrenska Academy, University of Gothenburg, Gothenburg, Sweden;; 3 Department of Oncology, Institute of Clinical Sciences, Sahlgrenska Academy, University of Gothenburg, Gothenburg, Sweden; and; 4 Regional Cancer Center West, Sahlgrenska University Hospital, Gothenburg, Sweden

**Keywords:** secondary cancer, α-particle, targeted α-therapy, human, astatine-211, radium-224

## Abstract

Intraperitoneal ^211^At-based targeted α-therapy (TAT) may hold great promise as an adjuvant therapy after surgery and chemotherapy in epithelial ovarian cancer to eradicate any remaining undetectable disease. This implies that it will also be delivered to patients possibly already cured by the primary treatment. An estimate of long-term risks is therefore sought to determine whether the treatment is justified. **Methods:** Baseline data for risk estimates of α-particle irradiation were collected from published studies on excess cancer induction and mortality for subjects exposed to either ^224^Ra treatments or Thorotrast contrast agent (25% ThO_2_ colloid, containing ^232^Th). Organ dosimetry for ^224^Ra and Thorotrast irradiation were taken from the literature. These organ-specific risks were then applied to our previously reported dosimetry for intraperitoneal ^211^At-TAT patients. **Results:** Risk could be estimated for 10 different organ or organ groups. The calculated excess relative risk per gray (ERR/Gy) could be sorted into 2 groups. The lower-ERR/Gy group, ranging up to a value of approximately 5, included trachea, bronchus, and lung, at 0.52 (95% CI, 0.21–0.82); stomach, at 1.4 (95% CI, −5.0–7.9); lymphoid and hematopoietic system, at 2.17 (95% CI, 1.7–2.7); bone and articular cartilage, at 2.6 (95% CI, 2.0–3.3); breast, at 3.45 (95% CI, −10–17); and colon, at 4.5 (95% CI, −3.5–13). The higher-ERR/Gy group, ranging from approximately 10 to 15, included urinary bladder, at 10.1 (95% CI, 1.4–23); liver, at 14.2 (95% CI, 13–16); kidney, at 14.9 (95% CI, 3.9–26); and lip, oral cavity, and pharynx, at 15.20 (95% CI, 2.73–27.63). Applying a typical candidate patient (female, age 65 y) and correcting for the reference population mortality rate, the total estimated excess mortality for an intraperitoneal ^211^At-monoclonal antibody treatment amounted to 1.13 per 100 treated. More than half this excess originated from urinary bladder and kidney, 0.29 and 0.34, respectively. Depending on various adjustments in calculation and assumptions on competing risks, excess mortality could range from 0.11 to 1.84 per 100 treated. **Conclusion:** Published epidemiologic data on lifelong detriment after α-particle irradiation and its dosimetry allowed calculations to estimate the risk for secondary cancer after ^211^At-based intraperitoneal TAT. Measures to reduce dose to the urinary organs may further decrease the estimated relative low risk for secondary cancer from ^211^At-monoclonal antibody–based intraperitoneal TAT.

## Visual Abstract

**Figure:**
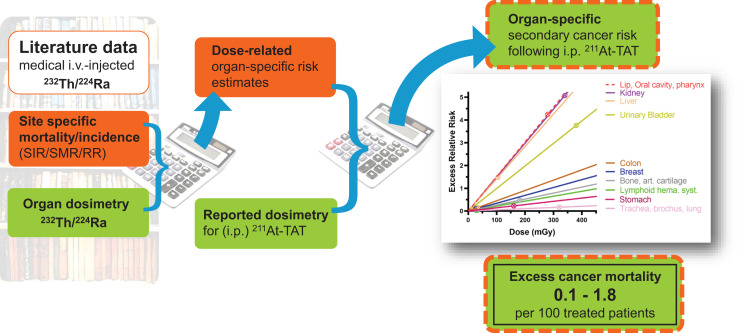

Radionuclides that emit α-particles are being evaluated for targeted α-therapy (TAT). However, estimates of long-term risks, such as for induction of secondary cancer, have not been a priority in the performed early-phase studies, probably because most patients considered for TAT at this early phase of drug development have late-stage disease for which treatment is not aimed at curing the patient.

The combination of high energy and short range makes α-irradiation most promising for delivering a high absorbed dose to target volumes of less than 1 mm^3^ (1). TAT is thus ideal for adjuvant therapy, that is, after primary treatment with surgery, radiation therapy or pharmacologic therapy, when patients are disease-free by objective measures but carry a statistical risk of recurrence. Since all use of radiation in medicine must be properly justified, the treatment benefit must outweigh any possible and probable risks. Such justification becomes more delicate for an adjuvant setting in which a proportion of the patients is already cured by the primary treatment.

Low organ-absorbed doses, well below estimated tolerance doses, were found in a phase I study with intraperitoneal delivery of therapeutic amounts of ^211^At conjugated to MX35 F(ab′)_2_ (^211^At-monoclonal antibody [mAb]) (2). In a study of 12 patients, including 4 patients with 6- to 12-y survival, no radiation-linked acute toxicity was observed and no other observable side effects were revealed (3). As the risk to induce secondary cancer by radiation can be calculated, the effective dose for this treatment has been published (4). Effective dose is, however, intended for application only in radiation protection and can at best provide a rough estimate of the long-term risk. For α-particle irradiation, a conservative radiation-weighting factor of 20 is applied, whereby risk might be overestimated. If this leads to delivery of overly cautious (e.g., lower) amounts of the therapeutic agent, the therapy results might be negatively affected. There is an obvious need to estimate the risk of induction of secondary cancers, particularly for planning further clinical studies in the adjuvant setting, in which long-term survival is expected.

In this work, we estimated carcinogenic risks for a novel TAT by comparing resulting organ-absorbed doses with the best data available on cancer incidence and mortality from long-term follow-up of patients who received α-emitting substances. By directly calculating risk after α-irradiation from the known long-term effects of other α-irradiations, the uncertainty involved in determining a radiation-weighting factor for α-irradiation is eliminated. Published organ-by-organ relative risks for secondary cancer after administration of the α-emitting compounds Thorotrast (5–9) and ^224^Ra (10–12) were used.

The aim of this work was to compile and evaluate published data relevant to the estimation of carcinogenic risk that could prove useful for justifying adjuvant intraperitoneal TAT. It might also serve as a reasonable method for estimating long-term toxicity for other TATs.

## MATERIALS AND METHODS

### Background Data

All data are from published studies or public registers in which no individual data can be distinguished. The ^211^At-mAb study was approved by the Regional Ethical Committee, and written informed consent was obtained (2).

Only studies with injected α-emitting solutions used for medical purpose were included, that is, excluding environmental exposure studies. Two types of studies were identified as suitable: follow-up studies of patients who received the ^232^Th-containing radiographic contrast medium Thorotrast (5–9) and patients who received ^224^Ra for the treatment of tuberculosis and ankylosing spondylitis (10–12). In all, 6 Thorotrast series (Table 1) and 2 ^224^Ra studies (Table 2) were identified. A small overlap of included subjects is reported from the Japanese autopsy study (Table 1) (8). We excluded data on lung cancer from the ^224^Ra studies and from the Thorotrast autopsy study. A lung cancer incidence or mortality lower than expected was reported and was thought to be due to less smoking among the treated patients because of the underlying medical condition, as discussed by Wick et al. Nekolla et al. (10*,*^11^), and Mori et al. (8).

**TABLE 1.** Background Thorotrast Data

Study	Design and statistical method	*n*	Sex (*n*)	Age (y)	Comparison group	Estimated dosage (mL)*	Exclusion criteria	Last follow-up	Follow-up time (y)	Alive at analysis	Excess cancers	Excess per 100 subjects	Expected no. of cancers
Becker (5)	Site-specific mortality/SMR	2,326	M: 1,717 (74%); F: 602 (26%)	Mean, 31	1,890	Mean, 21	Survival < 3 y^†^	2005	Mean, 34	2%	349	15	114
Travis (9)	Site-specific incidence/SIR and RR	1,204	M: 670 (56%); F: 534 (44%)	Mean, 35^‡^	1,180	Mean, 17	Survival < 2 y^†^	1992–1993	Mean, 22	7%	229	19	77
Travis (9)	Site-specific mortality/SMR and RR	446	M: 223 (51%); F: 216 (49%)	Mean, 41^‡^	212	Mean, 25	Survival < 2 y^†^	1992	Mean, 20	10%	50	11	18
dos Santos Silva (6)	Site-specific mortality/SMR and RR	1,096	M: 685 (62.5%); F: 411 (37.5%)	Mean, 35^‡^	1,014	Median, 20	Survival < 5 y^†^	1996	Mean, 15	6%	86	8	5
Mori (8)	Site-specific mortality/RR	262^§^	M: 262 (100%)	Mean, 25	1,630	Mean, 17	Survival < 10 y^†^	1998	—	7%	79	30	30
Kido (7)	Site-specific mortality	150^∥^	M: 150 (100%)	Mean, 22	1,144	10–19 mL (94%)	Dead before 1979	1998	—	12%	63	42	8
Mori (8)	Autopsy study	386	M: 348 (90%); F: 38 (10%)	Range, 9–47	172,350	Mean, 18	Survival < 10 y^†^	1998	Mean, 38; SD, 9	—	214	55	105

* Thorotrast dosage refers to volume (mL) of Thorotrast injected.

^†^ From Thorotrast injection.

^‡^ Estimated age.

^§^ 61 patients included in autopsy study (8).

^∥^ 69 patients included in autopsy study (8).

SMR = standardized mortality ratio; SIR = standardized incidence ratio; RR = relative risk.

**TABLE 2.** Background ^224^Ra Data

Study	Study design and statistical method	*n*	Sex (*n*)	Comparison group	Estimated activity (MBq)	Exclusion criteria	Last follow-up	Follow-up time (y)	Alive at analysis	Excess cancers	Excess per 100 subjects	Expected no. of cancers
Nekolla (10)	Site-specific incidence/SIR	682	M: 510 (75%); F: 172 (25%)	None	∼45	Age ≤ 20 y (full cohort, *n* = 899); lag time^†^	2007	∼55	6%	92*	13*	56*
Wick (11)	Site-specific incidence/SIR and RR	1,471	M: 1332 (91%); F: 139 (9%)	1,324	∼10 (0.17/kg)			Mean, 26	32%	23*	0.3	159

* Lung cases not included.

^†^ Nekolla (10) used 5- and 2-y lag times for solid tumors and hematologic malignancies, respectively.

SMR = standardized mortality ratio; SIR = standardized incidence ratio; RR = relative risk.

Mean age was not possible to calculate; reported as <20 y or >20 y.

To estimate risk, the standardized mortality ratio, standardized incidence ratio, or ratio between observed cases in the exposed group and a control group were extracted directly or calculated from the studies. Both sexes were included, whereas, when possible, individuals less than 20 y old were excluded. All 6 Thorotrast studies administered similar amounts of Thorotrast (Table 1), but the injected activity of ^224^Ra differed depending on the illness treated (Table 2).

In each study, excess relative risk per gray (ERR/Gy) was calculated for the organ sites at which absorbed doses were given (Supplemental Table 1; supplemental materials are available at http://jnm.snmjournals.org). The extracted observed and expected numbers of cancer cases or deaths per organ and study are presented in Supplemental Table 2. The absorbed doses used are presented in Table 3. The 95% CIs for standardized incidence ratio, standardized mortality ratio, and relative risk were calculated on the basis of a Poisson distribution for counts. For each analyzed organ site, appropriate studies were pooled and weighted with an inverse-variance approach (13). The metan macro for Stata/IC statistical software (release 16; StataCorp LLC) was used for the pooling calculations.

**TABLE 3.** Calculated Absorbed Dose Data and Background Natural Mortality

Organ/group of organs	ICD-10 code	Thorotrast (mGy)	^224^Ra, high (mGy)	^224^Ra, low (mGy)	^211^At (mGy)	Mortality per 100,000 in women aged 65+
Lip, oral cavity, pharynx	C00–C13	174	NA	NA	280	36,9
Stomach	C16	39	99	22	160	80
Colorectum, anal*	C18–C21	42	297	NA	36	397.2
Liver, intrahepatic bile ducts	C22	6,900	585	130	104	69.6
Trachea, bronchus, lung	C33–C34	1,094	99	22	320	570.5
Bone and articular cartilage	C40–C41	4,800	19,800	NA	182	3.8
Breast (female)	C50	NA	99	22	28	373
Kidney	C64–C65	45	333	74	340	67.3
Urinary bladder	C67	NA	99	NA	380	76.2
Lymphoid, hematopoietic, and related tissues	C81–C96	2,100	1,890	420	30	281.9

* Colon (C18–19), 70%; rectum (C20), ∼30%.

NA = dose data not available.

Thorotrast mean administered volume was 20 mL and mean exposure was 30 y using distribution data from Ishikawa et al. (16*,*^18^). ^224^Ra was 45 MBq (high) and 10 MBq (low), applied to distribution from Lassman et al. (19). ^211^At was 200 MBq/L in intraperitoneal infusion, with 24-h dwell time, from Cederkrantz et al. (4). Mortality data are from NORDCAN (14), weighted by natural mortality in age span (data from Statistics Sweden (15)).

The typical candidate being treated with ^211^At-mAb for ovarian cancer is a woman aged 50–60 y, and the lag period for long-term risk is assumed to be 10 y. Therefore, cancer site–specific mortality rates by 5-y age groups for women aged 65 y or older (65+) in Nordic countries from 2007 to 2016 were derived from NORDCAN (14). Weighting factors, based on the fraction of the population alive compared with the total population aged 65+, were applied to all-cause mortality rates to account for patients dying of other causes. All-cause mortality data were taken from Statistics Sweden (15) for Swedish women aged 65+. It was assumed that within each age group (65 to 85+), the mortality rate was constant when calculating the weighting factors for each site. The weighting factors can be seen in Supplemental Table 3.

$$\begin{array}{l} m_{w\;65+}=\underset{i=\mathrm{age group}\;}{\sum }m_{i}*w_{i} \end{array}$$ (Eq. 1)

Equation 1 calculates the weighted reference population mortality for ages 65+ (*m_w_*_ 65+_) for each site, with *m_i_* being the site-specific mortality for the age group and *w_i_* the fraction alive in the age group of the total population aged 65+.

### Dosimetry Data

Absorbed doses to liver after Thorotrast injection were derived from Ishikawa et al. (16), to bone and bone marrow from Kaul et al. (17), and to the remainder of the organs from Ishikawa et al. (18), with assumed distributions of radioactive daughters. Absorbed doses after ^224^Ra irradiation were taken from Lassman et al. (19). The resulting absorbed doses used are given in Table 3.

### Outcome

The excess mortality for each cancer site was calculated from the estimated ERR/Gy for this cancer site from the published epidemiologic studies and multiplied by the dose received in the corresponding organ (Gy) after a 200 MBq/L intraperitoneal treatment with ^211^At-mAb (4) and further multiplied by the weighted reference population mortality rate for this cancer site after 65 y of age (*m_w_*_ 65+_):

$$\text{Excess}\;\text{mortality}=\text{ERR}/\text{Gy}\times \text{Gy}\times m_{w\;65+}$$ (Eq. 2)

To calculate the total excess mortality from a treatment, the results from the different sites were summed.

## RESULTS

Published Thorotrast studies were based on 5,870 (69% male, 31% female) patients, and the ^224^Ra studies comprised 2,153 patients (86% male, 14% female). Thus, the total for both treatment cohorts was 8,023 patients (74% male, 26% female). The median follow-up time was 26 y (range, 15–55 y). In total, 1,638 observed cancer events were used, and the excess numbers of reported cancers were 1,071 and 119 for the Thorotrast and ^224^Ra cohorts, respectively. Details of number observed, number expected, risk ratio, and excess cancer per organ site and per study are presented in Supplemental Table 2. The included studies and patient characteristics are presented in Table 1 (Thorotrast) and Table 2 (^224^Ra). The calculated absorbed dose data per organ or group of organs are presented in Table 3.

### ERR/Gy

**FIGURE 1.**
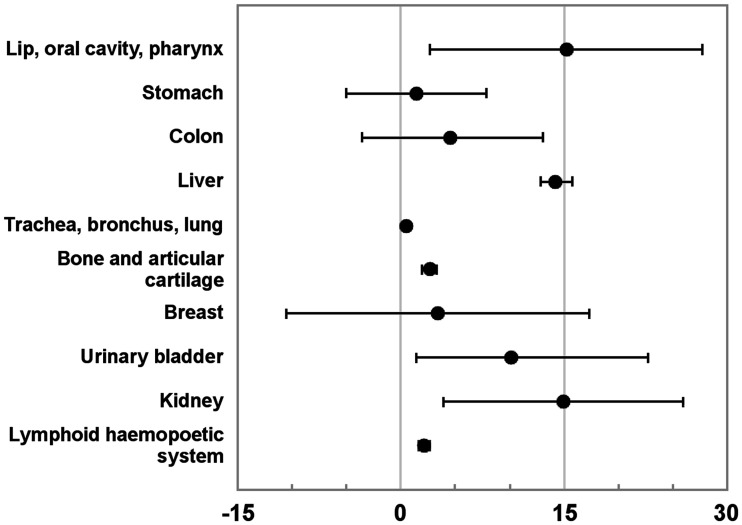
Pooled ERR/Gy for different organs or organ groups with 95% CI. Data for bladder represent only 1 background study (Nekolla et al. 2009 (10)).

The resulting pooled ERR/Gy for the 10 different organs or organ groups was calculated with 95% CI and presented in Figure 1. Generally, the 95% CI was wide, with lip, oral cavity, and pharynx being 15.20 (95% CI, 2.73–27.6); stomach, 1.43 (95% CI, −5.01–7.86); colon, 4.53 (95% CI, −3.95–13.01); breast, 3.45 (95% CI, −10.44–17.34); urinary bladder, 10.01 (95% CI, 1.39–22.28); and kidney, 14.93 (95% CI, 3.94–25.92), but was narrow for trachea, bronchus, and lung, at 0.52 (95% CI, 0.22–0.83); lymphoid and hematopoietic system, at 2.17 (95% CI, 1.68–2.66); bone and articular cartilage, at 2.63 (95% CI, 1.97–3.30); and liver, at 14.20 (95% CI, 12.82–15.57). Forest plots demonstrating the ERR/Gy from the respective individual studies and the resulting weighted overall ERR/Gy are found in Supplemental Figure 1 and Supplemental Table 1.

### Excess Mortality

The resulting excess mortality from cancer induction after a 200 MBq/L intraperitoneal treatment with ^211^At-mAb was estimated by multiplying treatment organ dose by the ERR/Gy and by the weighted natural mortality for a typical patient, that is, a woman 65 y old (Eq. 2). The total expected excess lifetime mortality from the treatment was 1.13 per 100 when the most solid background data were applied to derive ERR/Gy—that is, using both male and female observed and expected data. The influence of various assumptions and competing risk is presented in Table 4 and Supplemental Table 4. More than half the excess cancer mortality was from the urinary bladder (0.29) and the kidney (0.34). The lowest contribution to excess mortality per 100 was from bone and articular cartilage (0.002) and from the lymphoid and hematopoietic system (0.02).

**TABLE 4.** Influence of Various Assumptions and Competing Risk

		Excess cancer cases per 100 treated				
			FIGO stage			
Parameter	Adjustment in calculation or used background	Cancer-free	I	II	III	IV
Using ERR/Gy based on “male and female”*		1.13	0.90	0.64	0.25	0.11
Using ERR/Gy based on “female sex only”^†^		1.60	1.28	0.91	0.35	0.16
Age-dependence adjustment if low LET equals high LET*		1.11	0.89	0.63	0.24	0.11
Other second cancer risk after ovarian cancer primary*^‡^		1.53	1.24	0.87	0.34	0.15
Age (25 or 65 y) at time of treatment	ERR/Gy “male and female”*	1.24–0.90				
	ERR/Gy “female sex only”^†^	1.84–1.21				

* Calculations made with “male and female”–derived ERR/Gy in Supplemental Table 4.

^†^ Using ERR/Gy based on “female only.”

^‡^ Hazard risks (33), specified in Supplemental Table 4. No adjustment for decreased survival due to second primary cancer was performed.

Excess cancer cases per 100 treated are female patients, 55 y old at treatment. FIGO stage data are 10-y ovarian cancer relative survival according to International Federation of Gynecology and Obstetrics stage I–IV (0.80/0.57/0.22/0.11) (36). Influence of age at treatment (25 and 65 y) is as specified in Supplemental Table 5.

## DISCUSSION

With the introduction of TAT for clinical use, reliable risk estimations of long-term detriment, such as cancer induction, are needed to justify the procedure. The α-particles have a short pathlength and a high linear energy transfer (LET) that make them an ideal treatment for small-scale malignant disease. Adjuvant treatment in cancer aims at reducing the relapse rate for a cohort of patients subjected to treatment when compared with no treatment. Since only a fraction of these patients will relapse, it follows that the others are cured by the primary therapy. For the latter group, the adjuvant treatment will be of no benefit while carrying a possible risk. Therefore, a shared decision-making process is recommended when proposing an adjuvant therapy to the patient. The risks from all suggested treatments need to be disclosed and need be related to the expected gain from the therapy.

Estimation of risk is valuable for at least 2 reasons: to properly optimize and plan effect-finding studies and to provide patients with adequate information about possible benefits and risks. To state that the risks are unknown for a radiation-based therapy would not be correct or ethical. Although the risks are uncertain, some estimates would be a useful background for discussions with patients before they provide informed consent.

A recent study estimated excess cancer risk from a cohort of almost 150,000 patients after ^131^I treatment of well-differentiated thyroid cancer. A very small but statistically significant risk of second hematologic malignancy was found (20). That work initiated a debate on both the necessity of, and the difficulties with, performing such excess-risk estimates (21*,*^22^). It is evident that true risk can be assessed only after a long follow-up of patients exposed to a specific therapy, preferably in a randomized controlled trial.

We have previously used up to a 200 MBq/L intraperitoneal infusion of ^211^At-mAb in a phase I study, resulting in absorbed doses well below tolerance doses (using a relative biological effectiveness of 5) with low radiation-induced toxicity (2*,*^3^). Using biokinetic modeling, an activity concentration of 200 MBq/L was assumed sufficient to achieve eradicative absorbed doses to microtumors (23). When we applied the International Commission on Radiological Protection (ICRP)–recommended radiation-weighting factor of 20 for α-irradiation, the studied patients received an effective dose of 2.6 Sv (4) at this activity concentration. This would indicate a lifelong lethal cancer risk of around 10%. Effective dose should not, however, be used for any radiotherapy, as clearly stated by the ICRP itself (24). More specifically, the fundamental weaknesses of the effective dose as applied to α-irradiation has been thoroughly discussed (25). In the present work, we investigated whether the published literature contained relevant amounts of data to calculate risk directly, that is, data on epidemiologically derived carcinogenic risk after α-particle irradiation.

To do this investigation, we selected studies by focusing on long-term reports of carcinogenic risk after medical use of α-particle irradiation, that is, Thorotrast (^232^Th) (5–9) and ^224^Ra (10–12). These studies from multiple research groups contain well-documented radionuclide exposure to several thousand patients and include lifelong follow-up. Notably, most reported organ doses for ^232^Th and ^224^Ra are low and comparable to the organ doses received after adjuvant intraperitoneal TAT with ^211^At-labeled antibodies. Contributions from electrons and photons were considered negligible.

Thorotrast was a colloidal suspension of 25% ThO_2_ (including ^232^Th) used as an injectable contrast agent in the 1930s–1940s (26). The long biologic half-life resulted in lifelong irradiation (27), and lifetime doses of several gray (Gy) were received in reticuloendothelial organs, with a resulting clear excess risk of cancers (16). Approximately 5% of the ^232^Th will distribute to other tissues, with absorbed doses of 0.01–0.1 Gy (18), a level at which cancer excess is not always statistically significant, but this level is included in this combined analysis. The strength of the dose calculations for Thorotrast lies in the use of actual measured thorium concentrations, in several tissues, from a reasonable number of individuals, whereas the main uncertainty lies in estimating the contribution from ^232^Th daughters (16*,*^18^).

^224^Ra-radium chloride, as a component of Peteosthor, was used to treat bone tuberculosis or ankylosing spondylitis until the early 2000s (11). Its use for the treatment of children with bone tuberculosis was stopped in 1956 because of the reported growth retardation and excess occurrence of bone sarcomas (28). The amount of ^224^Ra radioactivity administered up to that time was approximately 50 MBq in the high-dose treatment. Thereafter, activity was reduced to about 10 MBq for treatment of ankylosing spondylitis in young adults. For the ^224^Ra dosimetry, we used data from Lassman et al. (19) that are based on the age-dependent biokinetic model for alkaline earth elements as described in ICRP publication 67 (*29*). In the current work, we have excluded patients younger than 20 y, but the mean age of the remaining patients is still comparatively low, at 37 y. The mean latency time for ^224^Ra-induced bone cancer was reported to be approximately 15 y (30). For other malignancies, the mean latency times were approximately 25 y, though presented with large uncertainty (10*,*^11^).

The ovarian cancer patients intended for an intraperitoneal ^211^At-mAb therapy have a median age of 63 y, which is clearly higher than those exposed to Thorotrast and ^224^Ra (33 and 37 y, respectively). For low-LET irradiation, the age dependence is not trivial (31). Although latency is generally long, the risk reduction by age at irradiation is not noticeable until approximately 65 y of age. The exceptions are breast and bone cancer, for which risk reduction is already seen at age 50 y at the time of irradiation (32). If an age dependence similar to that for low-LET radiation also applies to ^211^At-mAb–treated patients, the risk for breast and bone cancer (excess, 0.036 and 0.002 per 100 treated, respectively) could be reduced by a factor of approximately 2. However, since such data do not exist for high-LET irradiation, the main result of 1.13 excess cases of cancer per 100 treated is without any age correction. Moreover, a younger patient has a longer life expectancy than an older patient (of the same disease stage) and thereby a higher risk to be diagnosed with a secondary cancer. We accordingly adjusted the background mortality-rate data used, with a resultant risk decrease with higher age at treatment. In Table 4, only the excess cancer numbers for 25 and 65 y are presented, but details are provided in Supplemental Table 5.

In Table 4, the effects of making different assumptions or adding competing risks can be seen; all results are presented as number of excess cancer cases per 100 treated (with a 200 MBq/L intraperitoneal dose of ^211^At-mAb). For example, in the analyzed cohorts (Tables 1 and 2), female sex constituted approximately only 25% of all individuals. Two studies (5*,*^10^) contained some data grouped by sex. If only the female data were used to calculate the ERR/Gy, the number of excess cancer cases for a cancer-free woman of 55 y amounted to 1.60. We find this number more uncertain because the female-only–derived ERR/Gy is based on much fewer observed cancer cases with a resultant much wider 95% CI (Supplemental Table 4). Additionally, we demonstrated the effect of 2 kinds of competing risks. First, ovarian cancer stage correlates with mortality; that is, a patient at FIGO (International Federation of Gynecology and Obstetrics) stage IV is more likely to die from ovarian cancer than from development of a secondary cancer. Therefore, the excess cancer cases decrease when adjustment is made for survival of ovarian cancer as shown for International Federation of Gynecology and Obstetrics stages I–IV (Table 4). Second, patients surviving ovarian cancer are at increased risk of a second primary cancer, compared with the healthy population (33). To account for this increase, the excess cancer cases are multiplied by the hazard ratios for the risk to be diagnosed with a second primary cancer after an ovarian cancer diagnosis (33). This results in an increase from 1.13 to 1.53 per 100 treated “cancer-free” patients; no adjustment for decreased survival due to the secondary cancer was done (Table 4; Supplemental Table 4).

The use of cancer excess data after exposure to ^232^Th and ^224^Ra is not ideal because of the different half-lives and biologic distributions of these α-emitters. Also the microdistribution of decays within each organ will likely differ. On the other hand, they provide the best clinical data available for estimating long-term risk after α-particle therapies because the studies include solid data for some 8,000 patients with often lifelong follow-up. In our estimates, we have assumed that the risk is a linear function of the organ mean absorbed dose. This assumption implies a linear no-threshold model, which is reasonable if cancer induction originates from a single stochastic mutation induced by an α-particle traversing the cell nucleus (25). A deviation from linearity may be expected when high radiation doses are received in a short time, since an increased likelihood for cell death will reduce the cancer induction risk. For ^232^Th, several observations indicate a linear increase in risk with increasing dose. These observations include the fact that the liver receives the highest absorbed dose with a very heterogeneous dose distribution (34). Using a 2-mutation carcinogenesis model, the conclusion was that the excess absolute risk for liver tumors correlates linearly with absorbed dose (35).

The resulting highest excess cancer contributions were from the urinary bladder and kidney. However, diuretics and an open indwelling urinary catheter to reduce transit time can decrease the dose to the bladder and probably to the kidney. It is also likely that ^211^At-compounds with improved in vivo stability can reduce the risk to organs associated with uptake of free ^211^At.

## CONCLUSION

Relevant data in the published literature were found that allowed carcinogenic risk estimation. The results presented here should be viewed as a first estimate of long-term risk for cancer induction after intraperitoneal α-particle treatment. They carry uncertainties in both the presented excess cancer incidence and the dosimetry, while still representing the best risk estimations available today. Application of this method will strengthen the risk–benefit analysis for patient selection and provides valuable information on organs that might be expected to experience the largest effect of dose optimizations.

## DISCLOSURE

Lars Jacobsson, Tom Bäck, Sture Lindegren, Stig Palm, and Per Albertsson are cofounders of Aprit Biotech AB. Sture Lindegren is a cofounder of Atley Solutions AB. Per Karlsson and Erik Holmberg declare a research contract with PFS Genomics Inc. and PreludeDX. This work was supported by the Swedish Research Council, the Swedish Cancer Society, the King Gustav V Jubilee Clinic Research Foundation, the Swedish Radiation Safety Authority, the Gösta Miltons foundation, and grants from the Swedish state under an agreement between the Swedish government and the county councils (the ALF agreement [ALFGBG-435001]). No other potential conflict of interest relevant to this article was reported.

## Notes

KEY POINTS **QUESTION:** Can the risk of secondary cancer after an adjuvant intraperitoneal ^211^At-mAb–based therapy be estimated? **PERTINENT FINDINGS:** Using the organ dose from ^211^At, organ-specific risks were estimated from literature data on excess cases of cancer for subjects medically exposed to other α-particle emitters (^224^Ra or ^232^Th). The ERR/Gy was applied for patients receiving intraperitoneal ^211^At-TAT, and the total excess mortality might range from 0.1 to 1.8 per 100 treated, depending on various background data or assumptions of competing risks. **IMPLICATIONS FOR PATIENT CARE:** The estimation of carcinogenic risk is valuable for proper justification, optimization, and planning of effect-finding studies in forthcoming adjuvant therapy trials and to provide patients with adequate information about possible benefits and risks.
